# Selective Otolithic and Semicircular Canal Dysfunction: Insights from VEMP and vHIT

**DOI:** 10.3390/jcm15103944

**Published:** 2026-05-20

**Authors:** Pavol Skacik, Stefan Sivak, Egon Kurca

**Affiliations:** Neurology Department University Hospital Martin, Jessenius Faculty of Medicine Martin, Commenius University Bratislava, Mala Hora 4, 036 01 Martin, Slovakia; stefan.sivak@uniba.sk (S.S.); egon.kurca@uniba.sk (E.K.)

**Keywords:** vestibular dysfunction, otolith dysfunction, semicircular canal hypofunction, video head impulse test, vestibular evoked myogenic potentials

## Abstract

**Background/Objectives:** Vestibular evoked myogenic potentials (VEMPs) and the video head impulse test (vHIT) enable receptor-specific assessment of otolithic organs and semicircular canals. Their increasing use has revealed selective or apparently isolated vestibular abnormalities, although the clinical significance of these findings remains uncertain. This mini-review examines selective otolithic and semicircular canal dysfunction, with emphasis on diagnostic interpretation, methodological limitations, and future research needs. **Methods:** A structured narrative review of PubMed/MEDLINE and Scopus was conducted, focusing on studies reporting isolated, selective, or disproportionate vestibular abnormalities assessed by VEMPs and/or vHIT. Relevant original studies, case series, case reports, reviews, and diagnostic or consensus papers were considered. **Results:** Selective otolithic dysfunction may involve the utricle, saccule, or both and is often associated with imbalance, tilting, swaying, spatial disorientation, nausea, or postural instability. Selective semicircular canal dysfunction may involve one or more canals and may present with vertigo, dizziness, nystagmus, or gait instability. Similar VEMP and vHIT patterns occur across vestibular neuritis, Ménière’s disease, vestibular migraine, benign paroxysmal positional vertigo, bilateral vestibulopathy, superior semicircular canal dehiscence, vestibular schwannoma, central vestibular disorders, systemic diseases, and idiopathic presentations. **Conclusions:** Selective vestibular abnormalities should be interpreted as context-dependent laboratory findings rather than discrete disease entities. Their value depends on reproducibility, anatomical plausibility, clinical concordance, complementary testing, and longitudinal follow-up.

## 1. Introduction

The development of modern vestibular function tests, particularly vestibular evoked myogenic potentials (VEMPs) and the video head impulse test (vHIT), has substantially advanced the assessment of peripheral vestibular function. These techniques enable functional evaluation of all ten vestibular end-organs—the utricular and saccular maculae and the three semicircular canals in each labyrinth [1]. Consequently, dysfunction can now be characterised with greater anatomical and functional precision, broadening the recognised spectrum of vestibular disorders [2].

Targeted assessment of individual semicircular canals and otolithic organs has led to increased recognition of apparently isolated vestibular deficits [3,4,5,6,7]. In the context of otolithic dysfunction, this has prompted efforts to define diagnostic criteria for idiopathic isolated otolithic dysfunction and to standardise clinical and laboratory definitions [8,9]. However, it often remains unclear whether such abnormalities represent discrete disease entities, early or partial manifestations of broader vestibular pathology, transitional stages within progressive disorders, or findings related to increased detection sensitivity [10]. Variability in normative thresholds and the limited availability of longitudinal data further complicate clinical interpretation.

In this mini-review, we provide a clinically oriented narrative overview of current studies on apparently isolated or selective vestibular dysfunction assessed using VEMPs and vHIT. The emphasis is on interpretation of these laboratory-defined findings and on a practical clinical approach to patients presenting with such abnormalities. We discuss whether these findings may reflect focal end-organ or pathway-predominant dysfunction; partial manifestations of broader vestibular, central, or systemic disorders; or methodological variability, and highlight limitations of the current evidence base and priorities for future research.

## 2. Methods and Literature Search Strategy

This mini-review was designed as a structured narrative review. A literature search was performed in PubMed/MEDLINE and Scopus between December 2025 and March 2026 using the following search terms: “isolated vestibular dysfunction”, “selective vestibular dysfunction”, “isolated otolith dysfunction”, and “isolated semicircular canal dysfunction”.

Studies were considered eligible if they reported isolated, selective, or disproportionate otolithic or semicircular canal abnormalities assessed by vestibular evoked myogenic potentials (VEMPs) and/or video head impulse testing (vHIT), or if they provided relevant clinical, diagnostic, methodological, or pathophysiological context for interpreting such findings. Original studies, case series, clinically relevant case reports, reviews, and consensus or diagnostic papers were considered.

Studies were excluded if they did not address VEMP or vHIT findings, or did not provide clinically interpretable vestibular data. Because of the heterogeneity of the available literature, findings were synthesised qualitatively rather than quantitatively.

## 3. Isolated Otolith Dysfunction

### 3.1. Background and Diagnostic Considerations

The otolithic organs—the saccule and utricle—detect linear acceleration and gravitational forces [11]. Unlike acute unilateral semicircular canal dysfunction, which typically produces spinning vertigo and characteristic nystagmus patterns [12], isolated otolithic impairment has historically lacked a clearly defined clinical profile. Symptoms such as imbalance, tilting, or spatial disorientation are relatively non-specific and may contribute to diagnostic uncertainty.

Recognition of selective otolithic dysfunction was long limited by the absence of reliable clinical testing. Prior to the introduction of VEMPs in 1992, routine functional assessment of the maculae was not feasible [13]. The development of cervical VEMPs (cVEMPs) as markers of saccular pathway function and ocular VEMPs (oVEMPs) as markers of utricular pathway function, together with vHIT to assess concomitant canal involvement, established a practical framework for evaluating selective otolithic abnormalities [14].

Within this framework, several related terms should be distinguished. Isolated otolith dysfunction refers to a pattern in which VEMP findings suggest predominant otolith-mediated pathway impairment with relative preservation of semicircular canal function. Idiopathic otolith dysfunction refers to such a pattern when no underlying vestibular, central, systemic, structural, or methodological cause is identified [8]. By contrast, otolithic involvement within broader vestibular disorders refers to VEMP abnormalities occurring as one component of an established condition, such as Ménière’s disease, vestibular migraine, vestibular neuritis, bilateral vestibulopathy, or central vestibular disease. Thus, “isolated” describes the observed test pattern, whereas “idiopathic” should be reserved for cases remaining unexplained after appropriate clinical evaluation.

### 3.2. Mechanisms and Aetiology

Proposed mechanisms of isolated otolithic dysfunction include microvascular compromise, inflammatory processes, and degenerative changes affecting the macular receptors. However, the underlying pathophysiology often remains uncertain, and some cases are classified as idiopathic after other causes have been excluded [6].

Macular abnormalities have also been reported within established vestibular and systemic disorders, including Ménière’s disease, vestibular migraine [10], persistent postural–perceptual dizziness (PPPD) [15], obstructive sleep apnoea [16], vestibular neuritis, and structural lesions such as vestibular nerve or cerebellopontine angle tumours [17]. These observations indicate that VEMP abnormalities may reflect possible focal macular pathology, but may also represent partial otolithic involvement within broader peripheral, central, or systemic disease processes.

### 3.3. Clinical Presentation

Anatomically, dysfunction may involve the utricle, the saccule, or both maculae, with isolated saccular impairment reported less frequently [3]. Although VEMP testing may help differentiate utricular from saccular pathway involvement, its anatomical specificity is limited. Responses are influenced by reproducibility, age-related amplitude decline, muscle activation, stimulus parameters, and laboratory-specific normative thresholds. Moreover, cVEMP and oVEMP reflect complex otolith-mediated reflex pathways involving otolithic receptors, vestibular nerve divisions, brainstem circuits, ocular or cervical motor pathways, and descending modulatory influences. Therefore, abnormal VEMP findings indicate dysfunction within these pathways, but do not by themselves localise pathology exclusively to the saccule or utricle.

Clinically, otolithic dysfunction may present within an acute vestibular syndrome characterised by disequilibrium, postural instability, and gait disturbance rather than prominent rotational vertigo. Patients commonly report tilting, translation, swaying, or lateropulsion, reflecting altered graviceptive perception. Episodic lateral tilt has also been described [18]. Nausea and vomiting are frequent, and nystagmus may occur depending on asymmetry of involvement, although it lacks specificity [10,15].

In patients with chronic vestibular symptoms, where postural instability predominates, detection of otolithic abnormalities may expand the differential diagnosis and may complicate attribution of symptoms [19].

### 3.4. Interpretation and Diagnostic Considerations

Taken together, isolated otolithic dysfunction is best regarded as a heterogeneous clinical and laboratory spectrum rather than a discrete disease entity. It may include idiopathic cases, partial otolithic involvement within established vestibular disorders, and possible early or transitional manifestations of evolving pathology. Prospective longitudinal studies integrating symptom trajectories, vestibular testing, and mechanistic data are needed to clarify its natural history and clinical significance.

## 4. Isolated Semicircular Canal Dysfunction

### 4.1. Background and Diagnostic Considerations

This term is therefore used descriptively to denote a vHIT-defined pattern of canal-predominant hypofunction rather than a validated disease entity or anatomically isolated lesion. Semicircular canals encode angular acceleration. Compared with isolated otolithic dysfunction, selective canal involvement is more frequently described in the literature. Presentations range from single-canal hypofunction to multi-canal deficits [20]. In this review, isolated semicircular canal dysfunction is used as an operational, vHIT-based term referring to selective or disproportionate semicircular canal hypofunction, in which canal involvement predominates over otolithic abnormalities. From an aetiological perspective, these patterns do not represent a clearly bounded entity. They overlap with established conditions, including unilateral or bilateral vestibulopathies and other peripheral or central disorders, and should therefore be interpreted within the broader clinical context of patients presenting with vestibular symptoms [21,22].

### 4.2. Mechanisms and Aetiology

Multiple mechanisms may underlie isolated or canal-predominant semicircular canal dysfunction. Isolated posterior canal hypofunction has been increasingly recognised with the introduction of vHIT systems capable of reliable vertical canal assessment, and may occur in unilateral or bilateral forms [20,23,24]. Structural causes, such as congenital posterior canal aplasia associated with positional vertigo, have also been reported [25]. Involvement of the anterior and horizontal canals shows similar heterogeneity. Mechanical obstruction, such as canalith jam in benign paroxysmal positional vertigo (BPPV), can produce patterns consistent with focal canal hypofunction [26], and similar canalith-related phenomena have been described in anterior canal BPPV [27]. Superior canal dehiscence syndrome may also present with vHIT abnormalities involving one or more canals [28,29]. Together, these observations show that local end-organ disturbances may generate apparent canal-specific deficits without diffuse labyrinthine pathology.

Inflammatory mechanisms may also produce canal-predominant patterns. In superior vestibular neuritis, selective involvement of the superior vestibular nerve division may manifest as canal-specific hypofunction on vHIT [30]. Depending on the anatomical distribution of nerve injury, impairment may affect one canal or several [31], and concomitant utricular or saccular dysfunction may occur because of shared innervation and anatomical proximity [32].

Similar canal-predominant abnormalities may also occur as partial manifestations of broader disorders. These include Ménière’s disease, vestibular schwannoma, other vestibulo-cochlear disorders, systemic conditions such as atherosclerosis and autoimmune disease, bilateral vestibulopathy with distinct patterns of hypofunction or relative canal preservation [22,23], and neurodegenerative disorders, including spinocerebellar ataxia type 27B, which may produce vertical canal-dominant abnormalities [33]. Central vestibular syndromes can also mimic peripheral canal hypofunction [11,34,35], underscoring the need for careful clinical correlation.

### 4.3. Clinical Presentation

Clinically, isolated semicircular canal dysfunction may present with vertigo of variable onset, duration, and associated features, depending on the canal involved. Horizontal canal impairment due to canalith jam may produce spontaneous horizontal nystagmus with preserved VEMP responses, supporting focal canal involvement [26]. In anterior canal BPPV, spontaneous upbeating nystagmus has been described as a characteristic feature [27].

Posterior canal hypofunction may occur in acute, episodic, or chronic forms. In published cohorts, many cases have been classified as idiopathic, with a predominantly chronic course and gait instability as a leading complaint [23]. Dizziness or vertigo frequently accompanies these symptoms. In unilateral cases, ipsilateral vestibulo-cochlear deficits may suggest involvement of the inferior vestibular nerve. Downbeat nystagmus—including positional and head-shaking-induced forms—has been documented in this setting [23,36].

Bilateral posterior canal dysfunction is observed predominantly in older individuals and often coexists with sensorineural hearing loss and positional downbeat nystagmus. In idiopathic cases, this pattern has been proposed as a possible contributor to age-related disequilibrium (“presbyastasis”), although prospective studies are required to clarify its clinical and pathophysiological relevance [37].

### 4.4. Interpretation and Diagnostic Considerations

Evaluation of isolated semicircular canal dysfunction is inherently challenging. Overlap with other vestibulo-cochlear disorders is frequent [23], and it may be difficult to distinguish discrete entities from stages within a broader, potentially progressive disorder [22]. Importantly, true peripheral canal hypofunction should be distinguished from central vestibular disorders that may mimic peripheral vHIT abnormalities [11,34,35]. Peripheral canal hypofunction is more likely when reduced gain and corrective saccades are anatomically concordant with the affected canal or vestibular nerve division and consistent with the clinical syndrome. By contrast, central lesions may produce abnormal gain, corrective saccades, or complex patterns that do not respect a single canal or peripheral nerve distribution [11]. In addition, gain measurements may be influenced by technical factors such as goggle slippage, covert saccades, calibration variability, and examiner technique [38].

**Figure 1:**
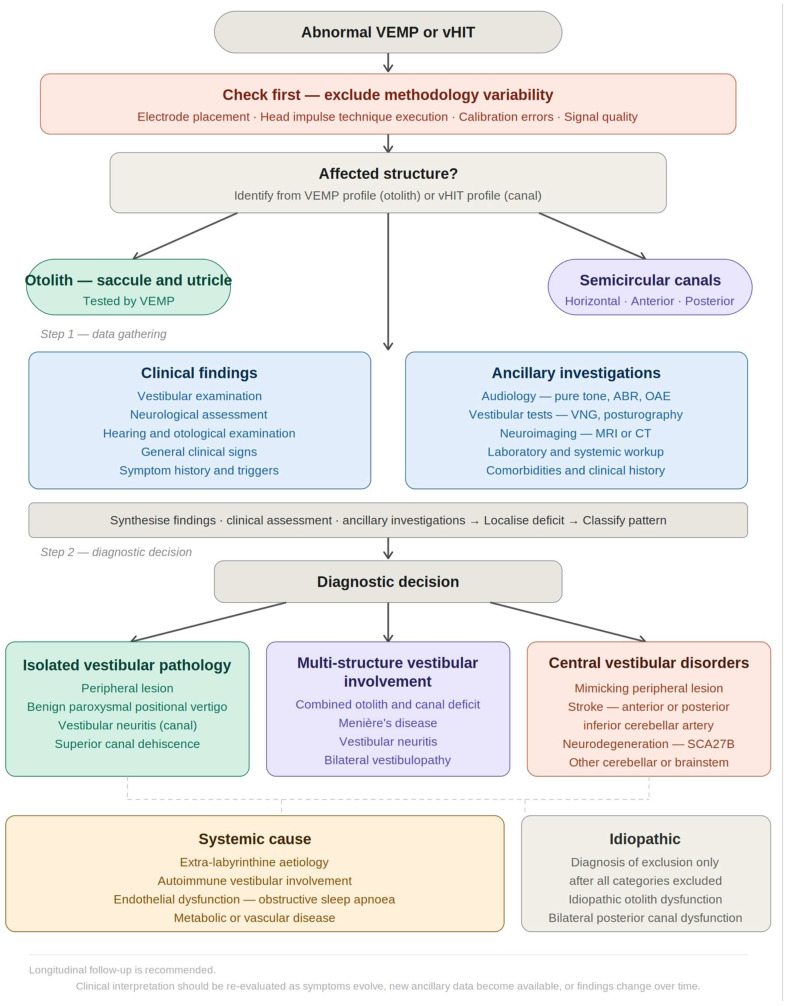
Proposed practical framework for interpreting abnormal VEMP and vHIT findings in clinical practice. Legend: Technical and methodological variability should be excluded before interpretation. Findings are then integrated with clinical assessment and ancillary investigations to classify abnormalities as isolated peripheral pathology, multi-structure vestibular involvement, central mimic, systemic cause, or idiopathic dysfunction as a diagnosis of exclusion. Longitudinal follow-up is recommended when uncertainty remains. *Abbreviations*: AICA, anterior inferior cerebellar artery; ABR, auditory brainstem response; BPPV, benign paroxysmal positional vertigo; CDP, computerised dynamic posturography; H/A/P, horizontal/anterior/posterior; OAE, otoacoustic emissions; OSA, obstructive sleep apnoea; PICA, posterior inferior cerebellar artery; PTA, pure-tone audiometry.

Accordingly, isolated or mild gain reductions should be interpreted in conjunction with the clinical presentation, nystagmus pattern, neurological examination, audiological findings, and, when appropriate, neuroimaging, to help differentiate peripheral canal hypofunction from central, degenerative, age-related, or methodological causes (Figure 1).

## 5. Discussion

Contemporary vestibular diagnostics, particularly VEMP and vHIT testing, have enabled identification of patterns of vestibular dysfunction that were previously difficult to detect [39]. However, canal- or macula-specific abnormalities do not necessarily indicate discrete end-organ diseases. They are better regarded as laboratory-defined patterns whose significance depends on clinical concordance, methodological reliability, and evolution over time.

Across published studies, it is useful to distinguish between focal end-organ or pathway-predominant dysfunction and selective abnormalities that represent partial manifestations of broader vestibular disease. First, focal processes, such as canalith mechanical obstruction or selective vestibular neuritis, may produce end-organ-predominant deficits [30]. In this context, the abnormality appears relatively localised to a specific semicircular canal, otolithic organ, or vestibular nerve division. However, even in these settings, involvement is not always restricted to a single receptor, and additional semicircular canal or otolithic abnormalities may reflect broader or evolving dysfunction [26]. Second, partial involvement within established vestibular or systemic disorders, including Ménière’s disease, bilateral vestibulopathy, vascular or autoimmune conditions, and neurodegenerative syndromes, may produce asymmetrical or receptor-predominant patterns that appear isolated at a single time point. These findings should be distinguished from truly focal dysfunction, because they may represent one component of a wider disease process rather than a discrete end-organ lesion. Third, physiological and technical variability, particularly in VEMP amplitude and vHIT gain interpretation, may contribute to apparent selectivity in the absence of stable pathology [35]. After these possibilities have been considered, idiopathic isolated vestibular dysfunction may be entertained as a diagnosis of exclusion [6,7,8,9,36].

The available evidence remains difficult to interpret because definitions, study designs, and testing protocols vary substantially [3,4,5,6,7,10]. The use of caloric testing, VNG, audiometry, posturography, neuroimaging, and follow-up is inconsistent across reports. This limits direct comparison between studies and reduces confidence that apparently selective findings always represent truly receptor-specific pathology.

Most available data come from case reports, small case series, retrospective cohorts, or cross-sectional analyses [3,6,40]. These designs are useful for identifying emerging clinical patterns, but they cannot reliably establish prevalence, natural history, prognostic value, or disease specificity [41].

Methodological factors are central to this uncertainty. VEMP responses are influenced by age, stimulus parameters, muscle activation, recording technique, and laboratory-specific normative values [42]. Similarly, vHIT gain may be affected by calibration, goggle slippage, examiner technique and methodology [43].

Disease specificity is also limited. Similar VEMP or vHIT patterns may occur in different disorders, while the same disorder may produce different test profiles depending on disease stage, lesion distribution, compensation, and methodology [39]. Thus, selective vestibular test abnormalities should narrow the differential diagnosis rather than serve as stand-alone diagnostic labels. For example, the overlap between vestibular migraine and Ménière’s disease illustrates this limitation. Recent systematic-review and meta-analytic evidence indicates that vHIT findings alone cannot reliably distinguish vestibular migraine from Ménière’s disease. These disorders therefore require diagnosis based on the full clinical context, including attack characteristics, auditory symptoms, audiometry, migraine features, and longitudinal evolution [44].

Clinically, dysfunction of any of the ten vestibular end-organs may produce symptoms because canal and otolithic inputs are integrated within central vestibular networks. Symptom expression is influenced by anatomical localisation, central compensation, adaptive mechanisms, comorbidities, age, and systemic factors [45,46]. Otolithic dysfunction may be associated with imbalance, tilting, swaying, translation, spatial disorientation, nausea, and postural instability, often without prominent rotational vertigo; however, these symptoms are non-specific, and nystagmus, when present, lacks diagnostic specificity [6]. Semicircular canal dysfunction typically presents with rotational vertigo of variable onset and duration, often accompanied by nystagmus patterns related to the affected canal and gait instability [40]. The temporal profile may range from acute and episodic forms to chronic presentations.

Taken together, patterns identified by VEMP and vHIT are best interpreted as a spectrum of laboratory findings with heterogeneous mechanisms rather than as discrete disease entities. Their clinical value is greatest when abnormalities are reproducible, anatomically plausible, concordant with symptoms and signs, and supported by complementary vestibular, audiological, imaging, or longitudinal data. In practice, interpretation should first consider technical and physiological confounders, then broader peripheral, central, systemic, and structural causes. A summary of reported VEMP and vHIT abnormalities across selected vestibular disorders, including their clinical correlates, is provided in Table 1.

Future research should prioritise prospective longitudinal cohorts incorporating standardised VEMP and vHIT protocols, explicit definitions of isolated or selective dysfunction, serial vestibular testing, imaging correlation, and clinically meaningful outcomes. Such approaches are needed to determine whether selective vestibular abnormalities represent stable focal deficits, early manifestations of broader vestibular disease, markers of progression, or incidental laboratory findings.

## 6. Conclusions

Contemporary vestibular testing, particularly VEMP and vHIT, has expanded recognition of canal- and macula-specific abnormalities. However, the current evidence base remains limited by heterogeneous definitions, variable testing protocols, small study populations, and a lack of longitudinal validation. These patterns are therefore best regarded as laboratory-defined findings with heterogeneous mechanisms rather than as discrete disease entities. Their interpretation should remain clinically contextual and, where possible, supported by reproducibility, complementary vestibular and audiological testing, imaging, and longitudinal follow-up. Further prospective studies using standardised protocols are required to clarify their natural history, diagnostic significance, and prognostic value.

## 7. Limitations and Future Directions

This review is limited by its narrative design and by the heterogeneity of available studies, including differences in diagnostic definitions, patient populations, vestibular testing protocols, and outcome measures. Most evidence comes from case reports, small case series, retrospective cohorts, or cross-sectional studies, which limits conclusions regarding prevalence, natural history, disease specificity, prognosis, and treatment response. Future prospective longitudinal studies using standardised VEMP and vHIT protocols, multimodal vestibular assessment, and clinically meaningful outcomes are needed to clarify whether selective vestibular abnormalities represent focal pathology, early or partial disease manifestations, technical variability, or incidental findings.

## Figures and Tables

**Table 1:** Disorders associated with VEMP and vHIT abnormalities and their clinical correlates.

Disorder	vHIT Abnormalities	VEMP Abnormalities	Clinical Picture	Clinical Interpretation of VEMP/vHIT Findings	References
Vestibular neuritis	Reduced gain in affected canal/canals, covert/overt saccades	cVEMP/oVEMP abnormalities depending on vestibular nerve division affected	Acute vertigo, nystagmus, imbalance	Help characterise total/superior/inferior vestibular nerve division involvement	[30,31]
Ménière’s disease	Normal, reduced, or enhanced gain; covert/overt saccades depending on disease stage	Reduced or enhanced VEMP responses depending on disease stage	Episodic vertigo, hearing loss, tinnitus, aural fullness	Stage-dependent	[47]
Vestibular migraine	Usually normal vHIT gain; reduced gain or corrective saccades reported only in a minority of patients	Amplitude and latency abnormalities reported in some patients	Episodic vertigo, migraine headache	Findings may indicate vestibular pathway involvement in some patients, but vHIT and VEMP generally in normal range	[48]
PPPD	Usually normal; abnormalities occasionally reported	Possible otolith dysfunction, isolated or with semicircular canal abnormalities	Chronic dizziness, postural instability	Abnormalities may reflect preceding or comorbid vestibular dysfunction	[17]
BPPV	Usually normal; reduced gain, canal-dependent differences, or corrective saccades may occur in selected cases, including canalith jam	Usually normal	Positional vertigo	Abnormal vHIT findings may suggest canalith jam or associated canal dysfunction, but typical BPPV is primarily diagnosed clinically by positional testing	[5,26]
Bilateral vestibulopathy	Bilateral reduced gain; diagnostic criteria include horizontal canal gain <0.6	Variable results; possible dissociation between VEMP and vHIT	Oscillopsia, gait instability, chronic dizziness	Bilateral horizontal canal hypofunction on vHIT; VEMP findings may reveal variable otolithic involvement	[22,49]
SSCD	May affect vestibular responses from all three semicircular canals, not necessarily only the superior canal	Enhanced cVEMP and oVEMP amplitudes, lower thresholds	Sound-induced vertigo, autophony	Enhanced VEMP responses and lower thresholds may support the diagnosis when consistent with symptoms and temporal bone imaging	[50,51]
Vestibular schwannoma	Variable semicircular canal gain reduction, corrective saccades	Abnormal VEMP latencies or amplitudes	Hearing loss, imbalance	Findings may reflect vestibular nerve involvement	[52]
Obstructive sleep apnoea	Usually normal	Possible abnormalities, reported more frequently than vHIT abnormalities	Non-specific dizziness	Reported abnormalities may suggest otolithic vulnerability, but their routine diagnostic role remains uncertain	[18,53]
Central vestibular disorders	Variable gain changes and corrective saccades; may mimic peripheral lesions	Variable findings reported	Central vestibular syndrome, cerebellar signs, other neurological features	Abnormalities may mimic peripheral dysfunction in some cases and therefore require correlation with neurological examination and neuroimaging	[11,35,54]

**Abbreviations:** BPPV, benign paroxysmal positional vertigo; cVEMP, cervical vestibular evoked myogenic potential; oVEMP, ocular vestibular evoked myogenic potential; PPPD, persistent postural–perceptual dizziness; SSCD, superior semicircular canal dehiscence; VEMP, vestibular evoked myogenic potential; vHIT, video head impulse test. **Note:** The interpretation column summarises how reported VEMP/vHIT findings may contribute to clinical reasoning. These findings should not be interpreted in isolation, but in relation to symptoms, clinical examination, audiological findings, imaging, and longitudinal evolution.

## Data Availability

No new data were generated or analysed in this study. All information discussed is derived from previously published studies cited in the reference list.
